# P26 Redefining youth engagement in AMR through an intervarsity training programme

**DOI:** 10.1093/jacamr/dlaf118.033

**Published:** 2025-07-14

**Authors:** Kenneth Chukwuebuka Egwu, Ibrahim Adamu

**Affiliations:** Antimicrobial Resistance Intervarsity Training Program, Enugu, Nigeria; Antimicrobial Resistance Intervarsity Training Program, Enugu, Nigeria

## Abstract

**Background:**

It is estimated that three people die every day due to antimicrobial resistance (AMR). Of these mortalities, the sub-Saharan African region bears the brunt of it. While there have been ongoing efforts to curtail antimicrobial resistance, youths, who are the major workforce in Africa are often neglected. As a way of redefining and strengthening youth engagement in AMR advocacy, we piloted an 8 month training programme, through support from the British Society for Antimicrobial Chemotherapy’s Stop Superbugs initiative, to educate the youth about the challenge, support the establishment of AMR clubs in tertiary institutions, and mobilize youths to conduct AMR awareness campaigns in underserved communities in Nigeria.

**Objectives:**

The programme aims to build the capacity of youth as future leaders to tackle the rising wave of antimicrobial resistance through training, community engagement, research, policy advocacy, and technological innovation.

**Methods:**

We conducted a 4 month training programme for 25 individuals from November 2024 to February 2025 as part of our ongoing 8 months intervarsity training programme. The participants were educated on key components of AMR such as mechanisms of resistance, antimicrobial stewardship, one health, infection prevention and control, behavioural change, community engagement, roles of young people in AMR advocacy, and storytelling for AMR. Additionally, we provided resources and technical support for AMR club registration in the participants schools. The teams were also supported with campaign materials and resources to conduct awareness activities within their communities, including schools, religious institutions, hospitals, and marketplaces.

**Results:**

The programme resulted in the establishment of five AMR clubs in five universities in Nigeria. The community engagement efforts reached 5692 students and teachers in schools, 3200 people in the marketplaces, 2050 people in churches and mosques, and 400 patients and caregivers in hospitals. The clubs have also grown into a network of 349 youth members joining to promote awareness about AMR nationwide.

**Conclusions:**

The results underscore the importance of young people in AMR advocacy. Through education and capacity building, the youths’ energy and vibrant passion can be harnessed to reach a wider audience and create more awareness about the challenge.

